# Assessing the accuracy of the third molar eruption as an indicator of adulthood: findings from a black South African sample using the Gambier method

**DOI:** 10.1007/s00414-025-03505-2

**Published:** 2025-05-19

**Authors:** Nikolaos Angelakopoulos, Shatakshi Sharma, Sudheer Babu Balla, Galina Zolotenkova, Stefano De Luca

**Affiliations:** 1 Department of Orthodontics and Dentofacial Orthopedics, University of Bern, Bern, Switzerland; 2 Independent Researcher, Dundee, Scotland, UK; 3 Dentistry and Oral Health, La Trobe Rural Health School, Bendigo, Australia; 4 Department of Forensic Medicine, I. M. Sechenov First Moscow State Medical University, Moscow, Russia; 5 Department of Biology of Organisms and Systems, Faculty of Biology, University of Oviedo, Oviedo, Spain

**Keywords:** Dental age estimation, Age of majority, Third molar eruption, Gambier et al. scoring system, South Africa

## Abstract

**Background:**

Age estimation is a critical aspect of forensic practice, often requiring straightforward, accurate, and precise dental methods employed by experts in various contexts where their expertise is needed. The third molar eruption analysis presents a practical approach. This study evaluates the Gambier et al. scoring system for assessing legal adulthood (18 years) through third molar observations in black South African subadults.

**Materials and methods:**

A retrospective analysis was conducted on 877 orthopantomograms (357 males and 520 females) of individuals aged 14 to 24 years.

**Results:**

The mean chronological age increased with the progression of stages (1 to 3) and phases (A to D) of the third molar eruption for both sexes. Our study revealed a strong association between advanced eruption phases and the likelihood of being 18 years or older. For instance, phase D (complete emergence in the occlusal plane) showed a high likelihood of individuals being 18 years or older, with 76% of males (32 out of 42) and 80.5% of females (66 out of 82) in this phase aged above 18.

**Conclusion:**

This technique, due to its ease of use, can provide useful preliminary information regarding the probable age of alleged minor asylum seekers. However, the results indicate that, in line with the minimum age principle, this method should be applied cautiously when determining adulthood, as the minimum age for both stage 3 of eruption and phase D is clearly below 18 years. Therefore, it should always be used alongside other validated methods recommended by international protocols and good practice guidelines to ensure reliability across different populations.

## Introduction

Age estimation in living individuals holds both legal and humanitarian significance, particularly in contexts such as criminal proceedings, immigration cases, competitive sports, and human trafficking. It is especially crucial in legal matters involving unaccompanied or asylum-seeking minors who lack proper identity documents, making the estimation of adolescents’ and young adults’ ages increasingly important [1–7]. In cases of doubt, forensic age estimations should ideally be conducted by forensic institutes that have the appropriate mandates and adhere to recommendations, such as those set forth by the Study Group on Forensic Age Diagnostics of the German Society of Forensic Medicine (AGFAD), last updated in 2008 [7, 8]. These guidelines specify that age estimation should include anamnesis and physical examination, radiography of the left hand, dental examination, and, when necessary, radiography or computed tomography of the clavicles. Accordingly, dental age estimation, involving direct examination of the oral cavity and an orthopantomogram (OPG), constitutes an integral and important component of the process for estimating biological age [7, 9].

It is well established that all teeth, except the third molars, complete their development between the ages of 12 and 14. Between the ages of 15.7 and 23.3 years, the third molars are typically the only teeth still undergoing development. Consequently, it has been suggested that the assessment of legal age should rely exclusively on the observation and measurement of third molar maturation [10].

Several forensic institutes across Europe routinely handle legal proceedings involving migrant minors without family references or alleged minor asylum seekers. Research consistently identifies individuals of Black African origin as a prominent demographic group among asylum seekers [11–17]. Various dental age estimation methods have been documented in the literature and tested on black African populations to estimate the age of majority [18–22]. However, due to legal time constraints, the personnel assigned to this task often include physicians and forensic anthropologists who lack specialized training in certain dental methodologies or the requisite expertise for their application in legal age estimation. Furthermore, in numerous instances, forensic odontologists with proficiency in this highly specialized domain of forensic practice are unavailable. This challenge is further compounded by the fact that forensic odontologists represent a niche scientific community and are not present in every country or city, further limiting access to expert assessments in legal proceedings.

In such exceptional circumstances, a user-friendly application that can be easily integrated into daily clinical practice, combined with a straightforward assessment of third molar eruption, should be employed to determine whether an individual is 18 years or older. A method meeting these criteria was introduced in 2019 by Gambier et al. [9], utilizing a French sample. This method focuses on the eruption of mandibular and maxillary third molars and is specifically designed to be user-friendly for physicians without specialized training in forensic odontology. They concluded that when both the maxillary and mandibular third molars are present in the oral cavity and have reached the occlusal plane, the probability of being over 18 years of age is very high for both males and females. This method has already been applied in previous studies, yielding applicable results in the initial phase of evaluating presumed minor subjects [23, 24]. In addition, Timme et al. [25] previously studied the efficacy of third molar eruption analysis in a sample of black South Africans using the Olze method [26]. They concluded that exclusive reliance on the evaluation of mandibular third molar eruption is insufficient for estimating the age of majority among black South Africans. Furthermore, they recommended that future studies focus on populations of African origin, where the development of permanent teeth exhibits distinct patterns. A critical factor in dental development is the timing of eruption [27], with African populations displaying a more advanced maturation rate compared to those of European origin [28]. These disparities in dental development highlight the necessity for population-specific dental standards, rather than relying on global norms.

In this context, our study had two main objectives: first, to evaluate the suitability of Gambier’s method [9] for estimating dental age in a sample of black South African sub-adults, and second, to assess the stages and phases of this method to determine whether an individual has reached 18 years of age based on the findings.

## Materials and methodology

### Materials

#### Sample

A sample of orthopantomograms (OPGs) from 877 healthy Black African subjects aged 14 to 24 years, comprising 357 males and 520 females, was analyzed. The data were collected retrospectively from the database of a private orthodontic clinic in Pretoria, South Africa, where OPGs were primarily taken for orthodontic treatment as part of the pre-treatment planning process. The subjects were registered anonymously, and their sex, date of birth, and date of X-ray acquisition were recorded for each OPG. Inclusion criteria required high-quality OPGs that displayed third molars in an intact condition. Exclusion criteria encompassed OPGs showing tumors, surgical materials, mandibular or maxillary fractures, gross pathology, a history of orthodontic treatment, or signs of infection in the third molar regions. Furthermore, OPGs of suboptimal quality, which impeded precise interpretation, as well as unclear radiographs affected by distortion, were excluded from the analysis. Likewise, individuals with a history of third molar extraction, those presenting with retained primary molars, and cases of all four third molars agenesis were also omitted. Socioeconomic status and specific ethnic groups were not assessed among the participants. The study was conducted on individuals who self-identified as black. None of the X-rays were taken specifically for the purposes of this research.

#### Data management

All third molars were numbered according to the two-digit FDI (Federation Dentaire Internationale) system [29]: 18 for the right upper third molar, 28 for the left upper third molar, 38 for the left lower third molar, and 48 for the right lower third molar. Each OPG was anonymized and assigned a unique identification number. Detailed information for each OPG, including identification number, sex, date of birth, date of radiograph exposure, chronological age, and stages/phases of each third molar, was meticulously recorded using Microsoft Excel 2016 for data management. The chronological age of each subject was calculated by subtracting the date of the X-rays from the date of birth and converting the result into decimal age. The study was carried out under the ethical standards laid down by the Declaration of Helsinki (Finland) and its later amendments [30].

### Methodology

#### Gambier et al. staging system

The Gambier et al. method [9] utilizes a three-stage scoring system. In stage 1, the third molar has an intact follicle, is positioned under the alveolar bone, and has not yet erupted. Stage 2 is characterized by a disrupted follicle and initial eruption, with one or more cusps breaking through the alveolar bone. Stage 3 indicates full eruption of the third molar, extending to the occlusal plane. For cases where OPGs depicted four assessable third molars, Gambier et al. [9] defined four phases as follows:

- Phase A: All four third molars classified as stage 1.
- Phase B: At least one third molar classified as stage 2.
- Phase C: At least one third molar classified as stage 3.
- Phase D: All four third molars classified as stage 3.

#### Image analysis and Intra-and-Inter-observer assessment

Each OPG was analyzed by a forensic odontologist (SBB) with nearly ten years of experience in dental age assessment. Unique numbering for each OPG facilitated a randomized and blinded examination process. Impaction was determined when a tooth deviated from its normal eruption path within the dental arch. To assess inter-examiner variability, a second examiner (SS), a junior forensic odontologist with less than one year of experience in dental age assessment, performed an additional evaluation of the selected OPGs. Furthermore, a subset of 100 OPGs was randomly chosen for re-evaluation by the first examiner (SBB) after a two-month interval to assess intra-examiner variability. The results presented in the study reflect the initial assessments conducted by the first examiner (SBB).

### Statistical analysis

The data were analyzed using IBM SPSS version 29.0 (SPSS Inc., Chicago, IL, USA). Intra- and inter-examiner agreement was assessed using Cohen’s kappa statistics. The initial analysis involved descriptive statistics to characterize each stage and phase of third molar eruption according to Gambier et al. [9]. Measures reported included mean, standard deviation (SD), median, lower and upper quartiles, as well as the range of minimum and maximum age values. A Student’s t-test was performed to compare mean ages across different stages of third molar eruption and to evaluate sex-based differences. A chi-square test was used to examine the association between age (≤ 18 years or > 18 years) and phase attainment. Subgroup analysis for age at the completion of the 18th year, considering both age and the Gambier et al. [9] phases of the third molar eruption, was conducted using a chi-square test with Bonferroni correction to adjust for multiple comparisons between the two age groups and the four eruption phases. The significance level was set at 5% (*p* < 0.05).

## Results

Table 1 shows the distribution of the overall sample by age and sex. Repeated scoring by the same examiner (intra-observer) revealed a very high agreement (Cohen’s kappa 0.95 (95% CI of 0.93–0.98), while inter-observer agreement was 0.91 (95% CI of 0.88–0.93), respectively.

**Table 1:** Age-and-sex-distribution of the total sample

Age group	South-Africa	
	Male	Female
14-14.9 years	74	86
15-15.9 years	54	93
16-16.9 years	52	76
17-17.9 years	46	69
18-18.9 years	44	41
19-19.9 years	34	53
20-20.9 years	23	35
21-21.9 years	14	30
22-22.9 years	9	15
23-23.9 years	7	22
Total	357	520

Table 2 provides an overview of the evaluability of third molars in the sample. Among the third molars, the maxillary molars (FDI 18 and 28) were most frequently evaluable, with only 2 to 3 cases of impaction, while the mandibular third molars (FDI 38 and 48) had higher rates of impaction, particularly tooth 38 with 59 impacted cases out of 877. Missing third molars were rare across all quadrants, supporting the sample’s comprehensive coverage of evaluable molars.

**Table 2:** Percentage of evaluable and non-evaluable third molars in the sample studied

		Country
		South-Africa
Tooth.18 Maxillary right third molar	Evaluable	858
	Missing	17
	Impacted	02
Total		877
Tooth.28 Maxillary left third molar	Evaluable	849
	Missing	25
	Impacted	03
Total		877
Tooth.38 Mandibular left third molar	Evaluable	793
	Missing	25
	Impacted	59
Total		877
Tooth.48 Mandibular right third molar	Evaluable	802
	Missing	21
	Impacted	54
Total		877

Table 3 presents the descriptive statistics of chronological age according to the Gambier et al. [9] eruption stages for each third molar, separated by sex. Mean chronological age increased progressively with each eruption stage, from stage 1 to stage 3, for all third molars and both males and females. For Stage 1, mean ages ranged from 15.72 years (mandibular right third molar, Tooth 48) in males to 16.38 years (maxillary right third molar, Tooth 18) in females. Stage 2 showed higher mean ages, with males averaging 17.88 years across most third molars, and females ranging from 17.71 to 17.92 years. Stage 3 was associated with the oldest ages, with mean ages between 19.15 years (Tooth 48) for males and 19.84 years (Tooth 18) for females. No statistically significant differences in mean ages between males and females were observed for each stage, though females tended to show slightly higher mean ages than males across stages.

**Table 3:** Descriptive statistics of the chronological age according to sex and gambier et al. eruption stages of all third molars (FDI notation)

	Stage	Male				Female			
		n	Mean	Min	Max	n	Mean	Min	Max
Tooth 18	1	189	16.19	14.01	23.39	259	16.38	14	23.27
	2	92	17.97	14.07	23.73	122	17.82	14.28	23.99
	3	69	19.59	14.30	23.85	127	19.84	14.35	23.79
Tooth 28	1	182	16.17	14.01	23.39	253	16.35	14	23.27
	2	91	17.88	14.07	23.47	124	17.92	14.28	23.98
	3	72	19.57	14.30	23.85	127	19.71	14.35	23.99
Tooth 38	1	148	15.74	14.01	21.96	212	16.12	14	23.27
	2	96	17.66	14.07	23.39	148	17.73	14.13	23.88
	3	75	19.29	15.30	23.47	114	19.66	14.28	23.99
Tooth 48	1	155	15.72	14.01	21.96	228	16.19	14	23.27
	2	90	17.88	14.07	23.39	134	17.71	14.13	23.99
	3	76	19.15	15.30	23.47	119	19.71	15.01	23.99

Descriptive statistics for the Gambier et al. eruption phases (A to D) by sex are summarized in Table 4. Mean chronological age showed a consistent increase from phase A to phase D for both sexes, indicating a clear relationship between the eruption phase and age. In Phase A, males had a mean age of 15.69 years (range: 14.01 to 21.96 years), while females had a mean of 16.06 years (range: 14.00 to 23.27 years). Phase B included slightly older individuals, with mean ages of 17.19 years for males and 17.27 years for females. In Phase C, mean ages increased further, with males at 19.09 years and females at 18.68 years. Phase D, which represents the final stage of eruption, was associated with the oldest individuals, showing mean ages of 19.29 years in males and 20.01 years in females.

**Table 4:** Descriptive statistics of the chronological age according to sex and gambier et al. eruption phases of all third molars (FDI notation)

Phase	Male				Female			
	*n*	Mean	Min	Max	*n*	Mean	Min	Max
A	127	15.69	14.01	21.96	183	16.06	14	23.27
B	83	17.19	14.07	23.39	139	17.27	14.13	23.88
C	48	19.09	14.30	23.47	54	18.68	14.65	23.99
D	42	19.29	15.30	23.13	82	20.01	15.01	23.79

Table 5 presents the distribution of the sample across age categories and Gambier stages (Stages 1 to 3) for each of the four third molars (teeth 18, 28, 38, and 48), divided by sex. Younger age groups (14–15.99 years) predominantly exhibit Stage 1 development across all four molars, while older age groups (18–23.99 years) show increasing frequencies of Stage 3, particularly in the maxillary molars.

**Table 5:** Distribution of the sample according to the age categories for each gambier stage studied for all four third molars (FDI notation)

Age Groups	Tooth 18			Tooth 28			Tooth 38			Tooth 48		
	Stage 1	Stage 2	Stage 3	Stage 1	Stage 2	Stage 3	Stage 1	Stage 2	Stage 3	Stage 1	Stage 2	Stage 3
**Males**												
14-14.99	66	5	1	64	6	1	65	7	0	65	7	0
15-15.99	41	9	2	40	10	2	32	16	3	37	11	4
16-16.99	27	19	5	26	20	5	22	19	7	24	15	11
17-17.99	24	15	6	23	12	7	14	18	4	15	16	5
18-18.99	15	15	14	14	17	12	10	9	19	10	13	13
19-19.99	5	14	14	4	11	18	2	11	19	1	13	19
20-20.99	7	8	8	7	8	8	2	9	8	2	8	9
21-21.99	2	4	8	2	3	9	1	5	6	1	5	7
22-22.99	1	1	7	1	3	5	0	1	7	0	1	6
23-23.99	1	2	4	1	1	5	0	1	2	0	1	2
**Females**												
14-14.99	76	6	2	77	7	1	73	10	1	75	10	0
15-15.99	67	17	8	65	18	7	57	23	9	62	18	11
16-16.99	43	24	7	41	23	9	36	27	6	39	25	7
17-17.99	28	30	11	26	30	13	17	33	15	19	33	13
18-18.99	15	12	13	15	9	15	8	16	12	9	14	12
19-19.99	13	17	21	12	16	23	9	18	19	11	16	20
20-20.99	6	5	22	6	7	20	4	10	16	4	8	17
21-21.99	7	3	20	6	5	19	5	6	16	5	4	18
22-22.99	2	1	11	2	2	9	1	2	9	1	2	10
23-23.99	2	7	12	3	7	11	2	3	11	3	4	11

Table 6 summarizes the age distribution of male and female subjects according to the four phases of the Gambier method (Phases A to D), reflecting the combined staging across all four third molars. The findings show a progressive shift from Phase A (all molars in Stage 1) in the youngest age group (14–14.99 years) to Phase D (all molars in Stage 3) in older adolescents and young adults. Notably, Phase D becomes more prevalent from age 18 onwards, with a marked increase among both sexes by ages 19–20 and beyond.

**Table 6:** Distribution of the sample according to the age categories for each gambier phase studied for all four third molars (FDI notation)

Age Groups	Males				Females			
	Phase A	Phase B	Phase C	Phase D	Phase A	Phase B	Phase C	Phase D
14-14.99	58	9	1	0	68	13	1	0
15-15.99	28	17	2	2	48	27	7	6
16-16.99	17	18	7	4	30	30	4	5
17-17.99	12	15	1	4	13	30	14	5
18-18.99	8	8	9	8	5	13	6	9
19-19.99	1	6	15	9	8	14	6	15
20-20.99	2	8	3	6	4	6	5	13
21-21.99	1	1	5	5	4	3	6	12
22-22.99	0	0	4	3	1	1	0	9
23-23.99	0	1	1	1	2	2	5	8

Table 7 displays the relationship between age (below or above 18 years) and eruption phases for both sexes. This analysis reveals a strong association between advanced eruption phases and the likelihood of being 18 years or older. In Phase A, 90.5% of males and 87% of females were less than 18 years of age. In contrast, Phase D showed a high likelihood of individuals being 18 years or older, with 76% of males and 80.5% of females in this phase aged above 18. These results demonstrate a clear association between third molar eruption stages and phases and chronological age, with the later eruption phases, particularly Phase D, strongly indicative of an individual being at least 18 years of age.

**Table 7:** Phase distribution (%) according to age (under or over 18 years) in both sexes

Age Classification	Male					Female				
	Phase A	Phase B	Phase C	Phase D	Total	Phase A	Phase B	Phase C	Phase D	Total
< 18 years	90.6	71.1	22.9	23.8	65	86.9	71.9	48.1	19.5	65.7
> 18 years	9.4	28.9	77.1	76.2	35	13.1	28.1	51.9	80.5	34.3
Total	100	100	100	100	100	100	100	100	100	100

**Fig. 1:**
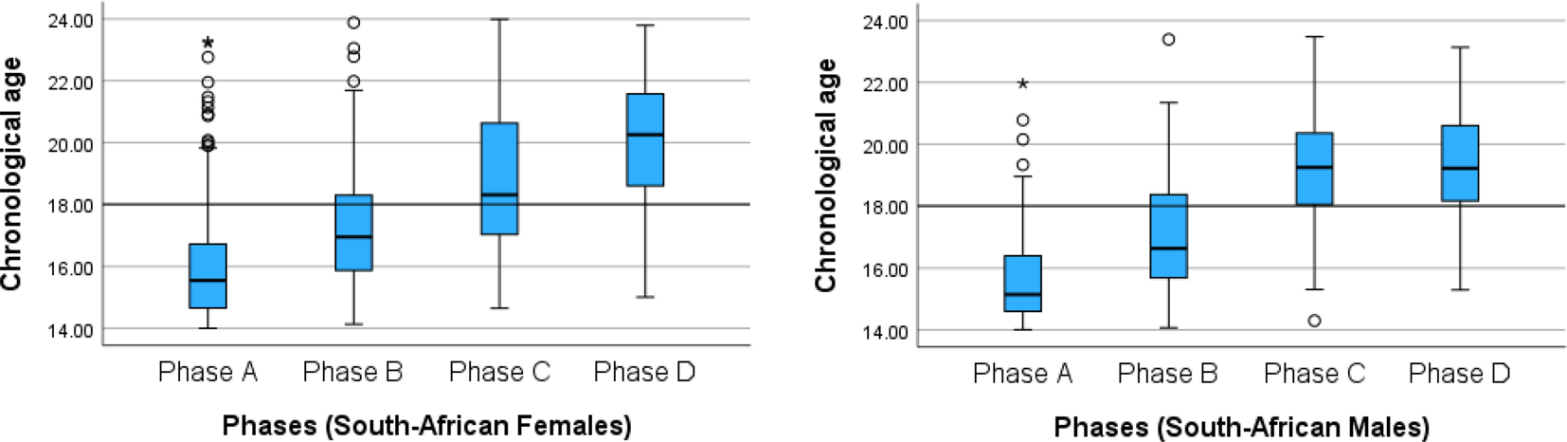
Box-plot graphical representation of age distribution for each third molar for males and females

Figure 1 illustrates the age distribution for each third molar stage across sexes in the South African sample, with distinct age clustering observed at each eruption stage. Males and females exhibited similar age distributions within stages, yet with a consistent tendency for females to show slightly higher mean ages than males, particularly at stages 2 and 3. These boxplots highlight a broad age range at each stage, with considerable overlap among stages.

## Discussion

Limited research exists on the temporal patterns of mandibular third molar eruption in black Africans for use in forensic age estimation [31–33]. In addition to recent research on third molar eruption, a study on the eruption of the first permanent molars in South African children was published in 1990 [34]. Furthermore, other studies on the eruption of all permanent teeth were conducted in various black populations in the 1970s [35–37]. All of these studies highlight the early eruption of permanent teeth in populations of African origin.

However, there is currently a lack of updated, comparable, and systematic studies on third molar eruption as a feature of forensic age estimation in different African populations. Cummaudo et al. [38] have emphasized the need for further research, particularly on the African continent, due to the extremely low rate of birth registration. For many Western and Central African countries (e.g., Liberia, Chad, Guinea-Bissau, Democratic Republic of Congo), there is currently no data on the rate of skeletal and dental maturation. Therefore, it is essential to validate methods developed for European populations, such as the Gambier method [9], in populations from countries with high numbers of undocumented minors, such as South Africa.

In the context of forensic and clinical literature, this is the first radiographic dental study to assess the suitability of Gambier’s method [9] for distinguishing between adults and minors in a sample of black South African subadults and young adults. In this study, we estimated the mean chronological ages at which the different stages of tooth eruption, as described by Gambier et al. [9], were observed.

Regarding intra- and interobserver agreement, the results indicate that the method is both repeatable and reproducible, making it an easy methodology to apply using radiographic support. Cohen’s Kappa values for intra-observer and inter-observer error were notably high, at 0.95 and 0.91, respectively. The Landis and Koch (1977) table can serve as a guide [39]. Consequently, the calculated Cohen’s Kappa coefficient of more than 0.90 indicates almost perfect reliability or agreement.

Regarding the eruption and mineralization of third molars, it has been observed that black African individuals exhibit accelerated development compared to those of European ancestry [40]. In fact, according to Olze et al. [32], subjects from South Africa have shown a faster third molar eruption rate compared to those from Asia (Japan) and Europe (Germany) in both biological sexes. In addition, Timme et al. [25], in a recent study using current reference data from a black South African population, observed that the eruption of wisdom teeth is typically completed around the age of 16 when Olze et al.‘s [26] method is applied. Unfortunately, this fact prevents the authors from using references from this population to estimate the legal age of 18 years. Kutesa et al. [33] in a study of Ugandan adolescents and young adults aged 10 to 20 years, applied Olze et al.‘s [32] method. They found complete eruption of the third molar as early as 13 years, which is consistent with earlier studies among Ugandans [41], Kenyans [42], Nigerians [43], and Indians [44].

This study verified that the eruption phases of the third molar, as described by Gambier et al. [9], are valid for estimating legal age in a sample of Black South African young adults. This analysis reveals a strong association between the likelihood of being 18 years or older and the corresponding phases of all third molars. In the original study, Gambier et al. [9] stated that individuals with all third molars in phase D have a relatively high likelihood of being 18 years or older. Chinni et al. [24] showed that 85.9% of males (220 out of 256) and 95.7% of females (176 out of 184) classified as phase D were indeed 18 years or older, suggesting that Gambier’s phase D of third molar eruption could be a useful indicator of reaching 18 years in the studied sample. Putul et al. [45] stated that third molar eruption could serve as a valuable age indicator in a sample of subjects from North-Eastern India, particularly when other reliable biological markers are unavailable during late adolescence. However, in this study, the method used to evaluate third molar eruption relied exclusively on clinical evaluation of the teeth, without the use of radiographic imaging.

When comparing the association between age (legal age of 18 years) and the stage of eruption of all third molars across both sexes, as observed in Chinni et al. [24] and in this study, it is noted that, in the South African population, the appearance of the different stages of the third molar is advanced by approximately one year in both females and males (see Table 3). These results are further supported by a study conducted on the South Indian population [46].

The minimum recorded age for the full emergence of third molars into the occlusal plane ranged from 14 to 15.1 years in females and from 14.01 to 15.30 years in males. Notably, a significant finding of this study is that the maximum observed age for stage 1 (unerupted) and stage 2 (partially erupted) third molars was 23.39 years in males and 23.27 years in females, while for stage 2, the upper limit reached 23.73 years in males and 23.99 years in females. Furthermore, based on phases A to C across nearly all quadrants, the maximum age for the complete emergence of third molars was 21.96, 23.39, and 23.47 years in males, and 23.27, 23.88, and 23.99 years in females, respectively. As the typical eruption range of the third molar spans from 17 to 25 years of age, our findings indicate that while the eruption process may commence early, its completion can be significantly delayed in certain cases, yet still fall within the normal range for this tooth. In such instances, the application of Gambier’s method [9] may present limitations due to the inherent uncertainty surrounding the full eruption process of the third molar.

Given the limited literature available, it can be stated that Gambier’s method [9] is applicable in all forensic contexts where estimating the age of presumed undocumented minors is necessary. However, it should be noted that, in accordance with the minimum age principle, its use is purely indicative. Additional tests are necessary to complement the information obtained, depending on the specific case and the available anatomical regions [8].

Additionally, since the Gambier method [9] involves the evaluation of all four molars, it may offer greater accuracy than the Olze method [26], which recommends analyzing only one third molar. However, in forensic practice, this does not mean one method excludes the other; both should be considered useful in the pre-assessment of the case. In fact, one of the first considerations is the likely geographic origin of the subject, followed by the subject’s biological sex. Therefore, since geographic origin is crucial for applying the most reliable method, it is essential that all methods be validated on as many population samples as possible. It is evident that, for reliable validation of the methodology, a balanced sample is necessary, considering factors such as age distribution within the proposed range (14–23 years) and the sex of the subjects. In other words, the distribution must be similar for each sex analyzed. However, complications arise when studying samples from countries where access to radiographic images is limited due to the economic constraints of dental patients or the lack of cooperation from local institutions, which may charge disproportionately high fees to access the samples. Even when such samples are available, the quality of the images is often poor.

### Limitations and future directions of this research

Therefore, as mentioned above, many studies conclude by recommending the validation of these methodologies—rarely used in forensic practice—on larger, more representative population samples. There is no doubt that this should be the primary objective of any forensic study. However, access to such samples is not always straightforward, which is a key reason why statistical analyses in several studies may appear inconclusive or unreliable. In this study, we have demonstrated the validity of Gambier’s method in a South African sample and confirmed its ease of application. With minimal training, the method can be applied effectively even by practitioners with limited experience. Even an inexperienced practitioner can use the method effectively. Therefore, the broader objective moving forward is not only to validate the method across additional population-specific samples, but also to develop tailored training programs for medicolegal professionals who wish to incorporate dental approaches, such as third molar analysis, into legal age estimation practices. The real future goal is not only to validate the method on additional specific samples but also to provide customized training sessions for medicolegal physicians who are interested in using these dental methods, which have proven useful for estimating legal age through third molar analysis.

## Conclusion

The results of this study suggest that Gambier’s classification for third molars may be useful only as an initial guideline for estimating the probable age of alleged minors in a black South African population. This approach could be particularly beneficial for forensic personnel who have limited experience with other recommended methods [8].

The results of this study indicate a strong association between advanced eruption phases (i.e., when all four third molars are present and have reached the occlusal plane) and the likelihood of an individual being 18 years or older. However, in accordance with the minimum age principle [8], the evidence does not conclusively confirm that the subject is definitively over 18 years of age.

Finally, this method is highly user-friendly and should continue to be an important component of forensic age estimation. However, since tooth eruption patterns may vary and can be subject to inaccuracies across different populations, caution must be exercised when estimating legal age. It is crucial to employ a combination of techniques, adopting a comprehensive approach to analyzing the third molar from both developmental and eruption perspectives.

## Data Availability

The data supporting this study’s findings are available on request from the corresponding author.
